# FungID: Innovative Fungi Identification Method with Chromogenic Profiling of Colony Color Patterns

**DOI:** 10.3390/pathogens14030242

**Published:** 2025-03-03

**Authors:** John Pouris, Konstantinos Konstantinidis, Ioanna Pyrri, Effie G. Papageorgiou, Chrysa Voyiatzaki

**Affiliations:** 1 Laboratory of Molecular Microbiology and Immunology, Department of Biomedical Sciences, University of West Attica, 12243 Athens, Greece; cvoyiatz@uniwa.gr; 2 Laboratory of Biology, Department of Medicine, Democritus University of Thrace, Dragana, 68100 Alexandroupolis, Greece; kostaskons94@gmail.com; 3 Department of Ecology and Systematics, Faculty of Biology, University of Athens, Panepistimioupoli, 15784 Athens, Greece; ipyrri@biol.uoa.gr; 4 Laboratory of Reliability and Quality Control in Laboratory Hematology (HemQcR), Department of Biomedical Sciences, School of Health and Care Sciences, University of West Attica, 12243 Athens, Greece; efipapag@uniwa.gr

**Keywords:** chromogenic, color pattern, fungi, FungID, fungi identification

## Abstract

Fungi play crucial roles in many ecosystems; however, traditional identification methods are often time- and labor-intensive. In this study, we introduce FungID, a pilot and novel deep learning algorithm, alongside its user-friendly software implementation, developed by analyzing various fungal species for identification based on chromogenic profiling of colony color patterns via a Convolutional Neural Network. Training and testing FungID upon a set of 269 images showed remarkable performance in terms of model robustness and classification efficacy. These findings demonstrate that FungID offers a potential method for rapid and reliable identification of fungal species through chromogenic profiling, providing additional tools to conventional techniques being employed in the fields of health, microbiology, biotechnology, and more. Our research underscores the promising role of deep learning algorithms in enhancing the understanding of the taxonomy and ecological functions of fungi that can be grown in pure cultures, while also emphasizing the importance of carefully assessing the scope and limitations of these methods.

## 1. Introduction

Fungi are among the most diverse groups of organisms, with a pivotal role in various ecological processes and numerous implications to human affairs. The identification of fungal species is critical for understanding their ecological impact, quantifying their influence on ecosystems, and assessing their potential in biotechnological applications. Traditionally, fungal identification relies on a combination of morphological, physiological, and biochemical features. The oldest and still widely used method is based on observing morphological features, including colony morphology, reproductive structures, and spore shape, size, ornamentation, etc., [1,2]. Detailed morphological characterization through light microscopy often allows for the recognition of distinct taxa [3].

Culture-based techniques involve isolating and cultivating fungi on selective and differential media to obtain pure cultures [4]. These methods, while accurate, are time-consuming and require expertise and equipment [5]. These resources are invaluable for mycologists and microbiologists working on fungi and their designation. But morphological identification is not always easy, especially for separating phenotypically similar species [6].

Molecular identification techniques have revolutionized the field of fungal taxonomy, offering more accurate and rapid methods for species identification, complementing traditional morphological approaches [7]. These techniques primarily rely on the analysis of genetic markers, which are specific regions of the fungal genome that provide distinctive characteristics for different species. The most widely used genetic markers include ribosomal RNA (rRNA) genes, such as the internal transcribed spacer (ITS) region, which serves as a universal barcode for fungal identification [8]. Another key marker is the large subunit (LSU) rRNA gene, which is often used for phylogenetic studies, providing greater resolution in complex fungal groups [9].

In addition to rRNA-based markers, protein-coding genes such as elongation factor 1-alpha (EF1-α) and RNA polymerase II (RPB2) have been utilized for higher resolution identification, especially in cases where closely related species are involved [10]. Whole-genome sequencing (WGS) is emerging as a powerful tool that can provide comprehensive data for precise fungal identification and phylogenetic analysis, offering insights into species-level differentiation and evolutionary relationships [11].

Polyphasic classification of fungi involves a structured approach used to identify and classify fungal species by combining different techniques and datasets [12,13]. Fungal classification is a complex task due to the vast diversity of species and the challenges in distinguishing them based on morphology alone [14,15]. Therefore, a polyphasic classification system incorporates various data, methods, and algorithms across different stages to improve accuracy and reliability in identifying fungi [13,14,15,16,17].

In the present study, an additional approach for fungal identification is proposed, namely chromogenic profiling, which analyzes the unique color patterns of fungal colonies growing on specific media. Chromogenic profiling discriminates taxa based on the analysis of fungal pigments through deep machine learning techniques [18]. The principle is based on the distinctive palette of pigments each fungus produces, which result in specific colony colors when grown in uniform environments [19,20]. Every color is unique and can be precisely identified through numerical systems like RGB. In these systems, colors are represented by combinations of values, each defining the intensity or properties of the components that constitute the color. For example, in the RGB model, every color is a unique blend of red, green, and blue values. This concept ensures that no two colors are exactly the same, providing a universal method to describe and reproduce colors with accuracy in digital and physical spaces [21]. Each color in fungal colonies is associated with the expression of specific genes under standardized conditions. These pigments can be constitutively produced or elicited by environmental signals, and their appearance and abundance vary among fungal taxa.

Chromogenic profiling systematically maps the color patterns of fungal colonies under different growth conditions to determine relationships between colony colors and identities. Chromogenic profiling also provides quantifiable color parameters that could be measured by digital imaging and image analysis software, therefore giving objective and reproducible results. This reduces subjectivity in interpretation and decreases the possibility for human errors, thus making fungal identification more reliable and robust. Although molecular techniques, particularly DNA sequencing, have focused on the taxonomy and identification of fungi in an effective way [15,22], they are still, per se, expensive and technically demanding [14]. Chromogenic profiling is a cost-effective complementary approach to molecular methods and therefore presents a pragmatic alternative.

Chromogenic profiling represents an innovative and promising approach to fungal identification, with broad applications in microbiology, ecology, biotechnology, and beyond. In this proof-of-principle study, we developed a pilot and novel algorithm as well as its user-friendly software implementation, named FungID, based on chromogenic profiling of colony color patterns, for efficient and accurate fungal identification. By deploying fungal pigment diversity, FungID provides a rapid, objective, and cost-effective means of differentiating fungal taxa, thereby advancing our understanding of fungal diversity and ecology.

## 2. Materials and Methods

### 2.1. Fungal Species Selection

For the experiments, ten fungal species were selected from the ATHUM Culture Collection of Fungi, of the National and Kapodistrian University of Athens Mycetotheca (Table 1). The selected strains include species of *Aspergillus* with similar color pallets, like *Aspergillus protuberus*, *Aspergillus nidulans*, and *Aspergillus flavusm*, or distinctive colors, like *Fusarium sporotrichioides* or *Alternaria alternata*, which were all identified using molecular techniques.

### 2.2. Media Preparation

Sabouraud Chloramphenicol Agar (Biolab Inc., H-1141 Budapest, Öv u. 43. Hungary) was used as the culture medium for fungal growth. This medium was prepared according to the manufacturer’s instructions. The agar contains peptones and glucose, optimized to support the growth of fungi, and is supplemented with chloramphenicol to inhibit bacterial contamination. After preparation, the medium was sterilized by autoclaving at 121 °C for 15 min and poured into sterile Petri dishes. Plates were allowed to solidify before inoculation.

### 2.3. Images

Images of fungal isolates were obtained by arranging Petri dishes in a semi-standardized manner, and photographed from the top in both light and dark backgrounds via a mobile cell phone, after being stabilized at a certain distance. The images, formatted as .jpg files, had resolutions of 3072 × 4080 (12.53 megapixels), 3120 × 4160 (12.97 megapixels), and 6144 × 8160 (50.13 megapixels).

### 2.4. Fungal Species Identification Algorithm and Implementation

**Figure 1:**
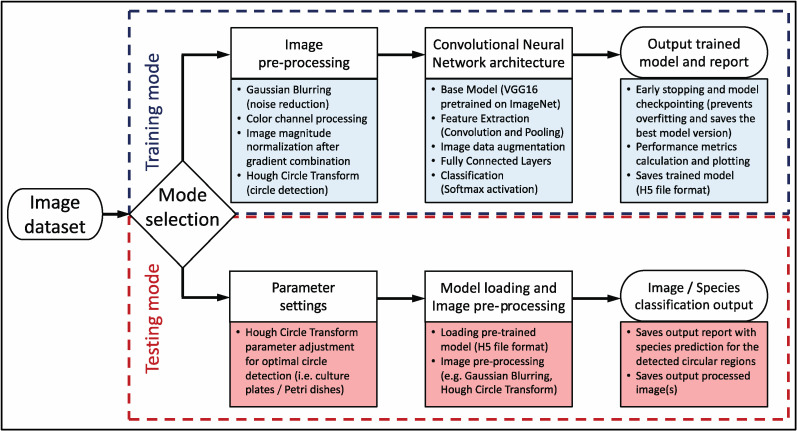
Workflow diagram of the FungID algorithm.

Our proof-of-principle algorithm for fungal species classification, namely FungID, is outlined in Figure 1, and was implemented using Python 3.8.5, with a combination of various libraries to handle image processing, neural network modeling, and the graphical user interface (GUI). The core libraries used here include tkinter [23] for the GUI, cv2 (OpenCV) [24] for image processing, numpy [25] for numerical operations, h5py [26] for handling HDF5 files, PIL (Pillow) [27] for image manipulation, and tensorflow.keras [28] for building and training the neural network model. Additional libraries such as matplotlib [29] were used for plotting training performance metrics, and threading was utilized to manage concurrent execution of tasks.

Technically, the core of the FungID algorithm is a Convolutional Neural Network (CNN), which was chosen over other approaches due to its ability to inherently capture spatial hierarchies present in image datasets, essential in distinguishing subtle differences in fungal colony morphology and color patterns [30,31]. The workflow of the algorithm begins with data preparation, where input data consisting of images of known fungal species are organized in a directory structure, with each subdirectory representing a different species. These images are preprocessed to ensure compatibility with the model in terms of format and size. The base model used is VGG16 [32], a popular deep learning model pretrained on the ImageNet dataset [33]. VGG16 is known for its deep architecture and ability to capture intricate patterns in images through its multiple layers of convolution and pooling operations. Convolutional layers apply convolution operations to the input image using a set of filters to create feature maps [31]. Each filter detects specific features, such as edges, textures, or patterns, within the image. Pooling layers, typically max pooling, are then used to reduce the spatial dimensions of the feature maps, retaining important features while reducing computational load [31]. Fully connected layers, including Global Average Pooling and Dense layers, add non-linearity and high-level reasoning to the model, allowing it to learn complex patterns [31]. The final dense layer uses a softmax activation function to produce probability scores for each class (fungi species), with the highest score indicating the predicted class [28].

OpenCV (Open Source Computer Vision Library) is employed in several preprocessing steps before feeding images into the CNN [24]. Gaussian blurring reduces noise and detail by applying a Gaussian filter to the input images. Color channel processing involves splitting the image into its RGB color channels and calculating gradients separately for each channel. These gradients are then combined to produce a normalized magnitude image, which emphasizes edges and features, making subsequent detection of circular regions (i.e., culture plates/Petri dishes) more effective. A key feature of OpenCV used in this process is the Hough Circle Transform [34], which detects circular regions of interest in the images. These regions likely contain fungi colonies on culture plates/Petri dishes. The main parameters for the Hough Circle Transform, which may be adjusted by the user, include dp (inverse ratio of the accumulator resolution to the image resolution), minDist (minimum distance between detected circle centers), param1 (higher threshold for the Canny edge detector), param2 (accumulator threshold for detected circle centers), minRadius (minimum detected circle radius), and maxRadius (maximum detected circle radius) [34]. The normalized magnitude image is passed to the Hough Circle Transform function to detect circles by identifying points corresponding to circle circumferences. Lastly, detected circles are outlined in green with red center points, and the regions inside them are cropped, resized, and passed through the CNN for classification.

As for the implementation of the proposed algorithm via Python, it consists of a user-friendly application with a GUI built with tkinter [23], providing several functionalities, like parameter adjustments for circle detection and an output console to display logs and results, as shown at Github (https://github.com/konskons11/FungID, accessed on 28 February 2025). The application GUI is divided into two modes, which may be enabled upon user selection: (i) “TRAIN MODE” and (ii) “TEST MODE”. During “TRAIN MODE”, an image dataset is loaded so as to train the software according to the input. The ImageDataGenerator [28] is used to augment the user input image dataset with various transformations to enhance model robustness. The training process incorporates early stopping and model checkpointing to prevent overfitting and to save the best performing version of the model. Progress is visually represented in the GUI with a progress bar. In “TEST MODE”, a pre-trained model may be loaded by the user, as well as the desired image(s) to be processed and classified (either single or multiple images). We highly recommend using our reference model for proper fungal classification as it comprises a substantial variety of fungal species across their developmental stages (file Ref_model.h5, https://github.com/konskons11/FungID/tree/main/Models, accessed on 28 February 2025). Detected circular regions are cropped, resized, and passed through the model to obtain classification probabilities. The results, including predicted fungal species and associated probabilities, are displayed in the GUI. Options to save classification reports and processed images are also provided.

In the context of our analysis, model performance was evaluated using a straightforward split of the data into training and test sets, with 70% allocated for training and 30% reserved for testing. To further assess the robustness of the model, we employed a 5-fold cross-validation technique due to our relatively small dataset. The dataset was divided into five equal parts, and in each of five iterations, one part was used as the test set while the remaining parts were used for training in 25 epochs. The model performance metrics accuracy, loss, validation accuracy, and validation loss were utilized and plotted per run for the 25 epochs. The classification results for each input image were stored after testing in separate files. In case multiple circular regions were detected within a single image, the classification results for the circle which best fitted the culture plates/Petri dishes were only extracted for downstream analysis. Confusion matrices were constructed for each run and overall and plotted as heatmaps via R programming language (version 4.2.3) in RStudio (build 382) [35,36]. Additionally, sensitivity, specificity, accuracy, precision (positive predictive value, PPV), negative predictive value (NPV), F1 score, and Matthews’s correlation coefficient (MCC) were calculated in R (version 4.2.3) [36,37]. Lastly, Receiver Operating Characteristic (ROC) curves and the area under the curve (AUC) were plotted for each fungal species via R programming language (version 4.2.3) in RStudio (build 382) [35,36].

## 3. Results

In our proof-of-concept study, we cultured a total of 10 fungi in three replicates belonging to 10 different species identified through molecular techniques, namely *Alternaria alternata*, *Aspergillus baeticus*, *Aspergillus caespitosus*, *Aspergillus flavus*, *Aspergillus nidulans*, *Aspergillus ochraceus*, *Aspergillus protuberus*, *Aspergillus sydowii*, *Fusarium sporotrichioides*, and *Penicillium chrysogenum*. We captured photographic images of these fungal cultures at various developmental stages, resulting in a total of 269 images. These images were then divided into five different subsets with minimal overlap, which were utilized for the training and testing of our proposed algorithm. Further information on the image dataset splitting process and the yielded classification results, after the implementation of the FungID algorithm, can be found in Github (file Fungi_prediction_results.xlsx, https://github.com/konskons11/FungID/blob/main/Models/, accessed on 28 February 2025).

### 3.1. Model Performance

**Figure 2:**
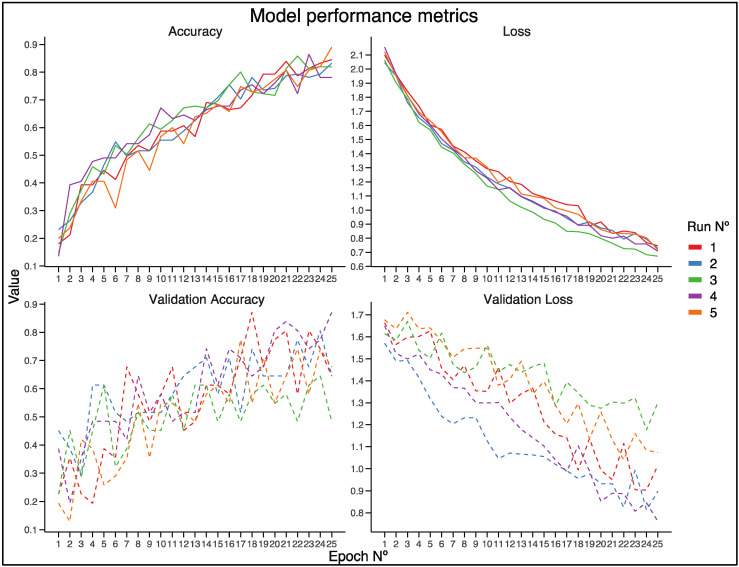
Model performance metrics over 25 epochs for five different iterations upon the corresponding image datasets, with each training run represented by its distinctive color (red, blue, green, purple, and orange). Top Left (Accuracy): This plot shows the training accuracy over epochs. All runs demonstrate an increasing trend in accuracy, with values ranging from approximately 0.2 to 0.9. Top Right (Loss): This plot shows the training loss over epochs. All runs exhibit a decreasing trend in loss, starting from around 2.0 and converging towards 0.7. Bottom Left (Validation Accuracy): This plot shows the validation accuracy over epochs. The validation accuracy fluctuates more compared to the training accuracy, with values ranging from approximately 0.2 to 0.8. Bottom Right (Validation Loss): This plot shows the validation loss over epochs. The validation loss generally decreases, with some fluctuations, starting from around 1.7 and converging towards 0.9. Overall, the consistent improvement in accuracy and reduction in loss across the five runs indicate the model’s robustness and reliability.

The model’s performance was evaluated by splitting our image dataset into 70% training data and 30% test data, with minimal overlap between the different subsets. The model was trained over 25 epochs, and the performance metrics, including accuracy, loss, validation accuracy, and validation loss, were recorded and summarized for each run. The first training run of our model started with a low accuracy of 0.1806 and a high loss of 2.0979. By the end of the training, the accuracy improved significantly to 0.8452, with a loss of 0.7464. The validation accuracy also showed substantial improvement from 0.2258 to 0.6452, with the validation loss decreasing from 1.6634 to 1.0158. As for the second run, the initial accuracy was 0.2323, with a loss of 2.0438. After 25 epochs, the model achieved an accuracy of 0.8323 and a loss of 0.7292. The validation accuracy increased from 0.4516 to 0.8065, while the validation loss reduced from 1.5700 to 0.8973. The third iteration of our training began with an accuracy of 0.1484 and a loss of 2.0612. By the final epoch, the accuracy rose to 0.8194, with the loss dropping to 0.6729. The validation accuracy improved from 0.2258 to 0.6452, and the validation loss decreased from 1.6148 to 1.2997. Starting with an accuracy of 0.1355 and a loss of 2.1547, the fourth training run ended with an accuracy of 0.7806 and a loss of 0.7089. The validation accuracy increased from 0.3871 to 0.8710, and the validation loss decreased from 1.6500 to 0.7603. Lastly, for the fifth and final training run, the initial accuracy was 0.2000, with a loss of 2.1211. By the end of the training, the model achieved an accuracy of 0.8903 and a loss of 0.7195. The validation accuracy improved from 0.1935 to 0.6774, while the validation loss reduced from 1.6771 to 1.0742. The detailed plots of these metrics can be seen in Figure 2.

### 3.2. Confusion Matrix

**Figure 3:**
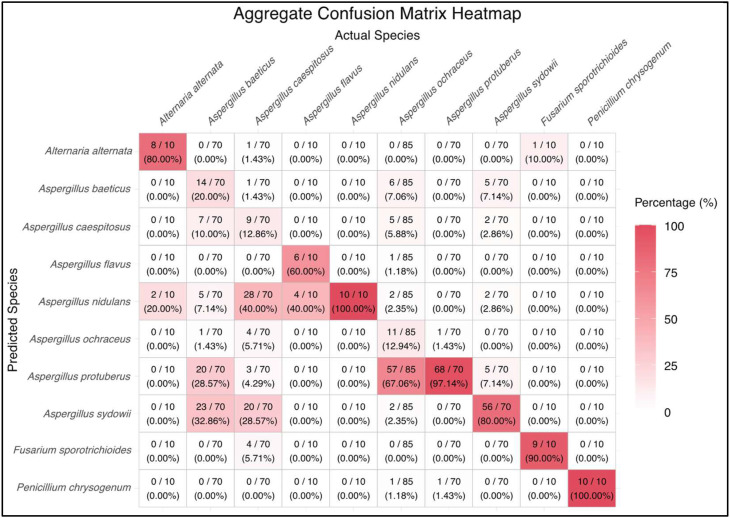
Aggregate confusion matrix plotted as heatmap, containing the results yielded after the five image classification testing runs. This heatmap illustrates the performance of the classification model in identifying various fungi species. The rows represent the predicted species, while the columns represent the actual species. Each cell shows the number of correct and incorrect predictions along with the corresponding percentage. The color intensity indicates the percentage of predictions, with darker shades representing higher percentages. Correct predictions are located along the diagonal, while misclassifications are off-diagonal.

The overall classification efficacy of the model was assessed using an aggregate confusion matrix, which provided a detailed breakdown of true positive, false positive, true negative, and false negative classifications for each one of the 10 examined fungi species. The confusion matrix was plotted as a heatmap (Figure 3), enabling a visual assessment of the model’s accuracy and misclassification rates. The diagonal elements of the heatmap represent the correct classifications, where the predicted species match the actual species. As shown in Figure 3, the model yielded almost a perfect score, ranging from 80% up to 100% accuracy for the species *Alternaria* sp., *Aspergillus nidulans*, *Aspergillus protoberus*, *Aspergillus sydowii*, *Fusarium* sp., and *Penicillium* sp., demonstrating its ability to distinguish these species effectively. However, notable underperformance and misclassifications were also observed, as in the case of *Aspergillus ochraceus*, which was incorrectly classified as *Aspergillus protoberus* 67.06% of the time.

The classification efficacy of the model was also evaluated using various metrics, extracted from the generated confusion matrices, including Sensitivity, Specificity, Accuracy, Precision (Positive Predictive Value—PPV), Negative Predictive Value (NPV), F1 score, and Matthews’s correlation coefficient (MCC). Overall, these metrics were calculated for each of the fungal species analyzed across the five testing runs. The results showed variability in the model’s performance across different species. Sensitivity ranged mainly from 80.00% to 100.00%, indicating the model’s ability to correctly identify true positives. Specificity values also fell in approximately the same range (75.36–99.75%), reflecting the model’s ability to correctly identify true negatives. Accuracy, which measures the overall correctness of the model, varied from 79.04% to 99.52%. Precision (PPV) values, which indicate the proportion of positive identifications that were actually correct, ranged from 18.87% to 85.71%. NPV values ranged from 81.41% to 100.00%, representing the proportion of negative identifications that were correct. The F1 score, which combines precision and recall/sensitivity, returned values between 0.19 and 0.91, while MCC values, which take into account true and false positives and negatives, thus providing a more balanced measure, ranged from 0.14 to 0.91, with an average of 0.54. The detailed values of these metrics are summarized in Table 2.

### 3.3. ROC-AUC Curve

**Figure 4:**
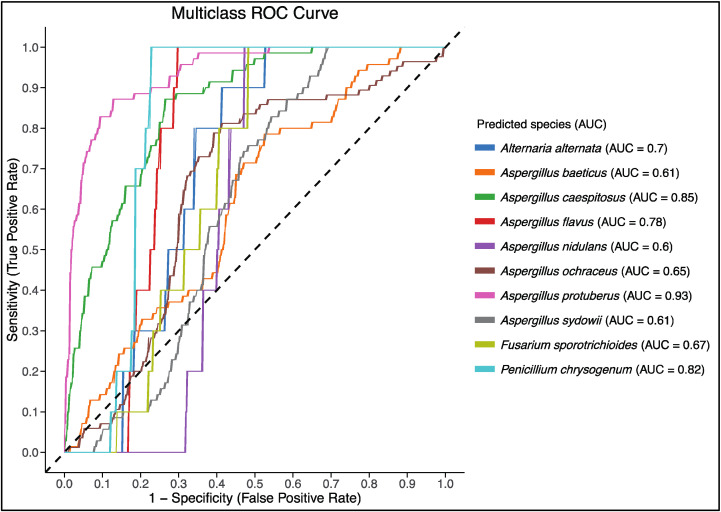
Multiclass ROC-AUC curve for all examined fungal species and testing runs in this study. Each curve represents a different fungal species, with its distinctive color and corresponding Area Under the Curve (AUC) values provided in the legend. The dashed diagonal line represents the performance of a random classifier (AUC = 0.5).

Last but not least, the Receiver Operating Characteristic (ROC) curve and the Area Under the Curve (AUC) values were utilized to assess the model’s diagnostic ability across all species and runs. The multiclass ROC-AUC curve provided insights into the trade-off between sensitivity (True Positive Rate) and specificity (False Positive Rate) for each species. The AUC values for each species indicated the model’s ability to correctly classify them. As illustrated in Figure 4, the model demonstrated varying performance across different species. Noteworthy, *Aspergillus protuberus* achieved the highest AUC value of 0.93, indicating excellent classification performance. In contrast, *Aspergillus nidulans* had the lowest AUC value of 0.60, suggesting relatively lower classification accuracy.

## 4. Discussion

This study presents a fungal species classification algorithm and its user-friendly application in pilot form, called FungID, leveraging a combination of advanced machine learning techniques and accessible GUI design for every user. The proposed algorithm, implemented in Python, integrates a range of libraries to ensure robust performance and ease of use. The application features dual modes for training and testing, providing built-in functionalities for data augmentation, early stopping, and model checkpointing. These features are critical for optimizing the training process and preventing overfitting, thereby enhancing the model’s robustness and reliability. A key strength of our FungID tool lies in its user-friendly GUI compared to the limited state-of-the-art methods for fungi identification [18], allowing users to interact with the algorithm seamlessly and proceed to immediate practical applications. The GUI offers functionalities such as parameter adjustments for circle detection, real-time monitoring of the training process through a progress bar, and direct visualization of classification results. The design makes the application accessible to both researchers and practitioners, even those with limited technical expertise. Users can easily switch between “TRAIN MODE” and “TEST MODE”, load pre-trained models, and process new images, thus enabling widespread adoption and practical application in various settings.

Despite the classification efficacy of the FungID algorithm, as demonstrated in the Section 3, several critical observations and potential improvements warrant discussion. Firstly, the model’s overall performance, as indicated by the aggregate confusion matrix and classification metrics, reveals notable strengths and some limitations. The high accuracy and sensitivity rates for species such as *Alternaria alternata*, *Aspergillus nidulans*, *Aspergillus protuberus*, *Aspergillus sydowii*, *Fusarium sporotrichioides*, and *Penicillium chrysogenum* underscore the model’s ability to distinguish these species effectively. Many of these fungi, including species in *Aspergillus*, *Alternaria*, *Fusarium*, and *Penicillium*, are of medical importance due to their potential to cause infections, particularly in immunocompromised individuals. The ability of our tool to accurately classify these species can be crucial for prompt diagnosis and treatment, potentially improving patient outcomes. However, the significant misclassification of *Aspergillus ochraceus* as *Aspergillus protuberus* highlights an area for improvement. This misclassification suggests that the model may struggle with species that have similar morphological features, pointing to the need for a more diverse and bigger training dataset or enhanced feature extraction techniques as pertains to the algorithm’s CNN architecture.

Moreover, the variability in metrics such as precision, recall, and MCC (Matthews Correlation Coefficient) across different species indicates that the model’s performance is not uniformly robust. MCC, in particular, provides a comprehensive measure that considers true and false positives and negatives, offering a balanced evaluation even for imbalanced datasets. In our study, there were cases, such as *A. alternata* and *P. chrysogenum*, which returned near-perfect precision and high MCC values, indicating accurate and reliable predictions, while others like *A. ochraceus* exhibited lower scores. This disparity again highlights that certain species may be underrepresented in the training dataset, leading to a reduced ability of the model to generalize well with new samples of these species. The lower precision for species like *A. ochraceus* suggests that the model often incorrectly predicts other species as *A. ochraceus*, leading to a higher false positive rate. Similarly, lower MCC values for these species indicate that the model’s predictions are less reliable when considering the balance between true and false positives and negatives. This imbalance could stem from the limited number of samples for certain species in the training dataset, which skews the learning process and affects the model’s ability to recognize and classify these species accurately. To address this issue, future work should focus on expanding the training dataset to include a more diverse range of species for each genus. This could involve collecting additional images representing various developmental stages, environmental conditions, and morphological variations. By enhancing the dataset’s diversity, the model can learn more generalized features that are representative of each species, improving its ability to accurately classify new samples [38].

The ROC-AUC analysis further corroborates these findings, with AUC values ranging from 0.60 to 0.93. This wide range highlights the variability in the model’s ability to distinguish between different species. The lower AUC for *Aspergillus nidulans*, which stands at 0.60, suggests that the model’s diagnostic ability is less reliable for this species compared to others. Several factors could contribute to this lower AUC, including dataset imbalance, where *A. nidulans* may have fewer samples compared to other species, leading to inadequate representation during training. Additionally, feature overlap between *A. nidulans* and other species could confuse the model, making it challenging to differentiate between species with similar morphological traits. Another contributing factor could be insufficiently nuanced feature extraction methods, which might fail to capture the subtle differences necessary for accurate classification, so addressing these issues may involve implementing more advanced techniques on the pilot form of the FungID algorithm.

As for the algorithm’s design and intelligence, CNNs seem to be the optimal approach for fungal identification through chromogenic profiling due to their ability to capture spatial hierarchies and local features in image data. According to the literature, alternative methods like Artificial Neural Networks (ANNs) or Support Vector Machines (SVMs) fall short in effectively handling spatial relationships in images, which is crucial for distinguishing subtle differences in fungal morphology and color patterns [39,40]. While ANNs are flexible and SVMs are powerful for small datasets, neither match CNNs’ efficacy in image classification [40]. Moreover, CNNs’ inherent translation invariance and availability of pre-trained models such as VGG-16 [32] make them superior for this task [41]. Thus, selecting CNNs ensures the highest classification accuracy and efficiency for fungal identification.

Nevertheless, CNNs have their own limitations and disadvantages, which majorly stem from overfitting issues due to small-sized datasets and vulnerability to adversarial attacks [42,43], potentially compromising the reliability of the trained models with subsequent underperformance. To enhance the robustness of our trained model and address these CNN issues in our research, we implemented strategies like (1) Data Augmentation, so as to artificially expand our dataset by rotating, scaling, flipping, and color jittering the input images to ultimately help the model generalize better after exposing it to a wider variety of samples derived from the original images [44], and (2) Cross-Validation, using a 5-fold cross-validation approach in order to ensure that the model’s performance is consistent across different subsets of our input image dataset [45]. In this pilot study, while our primary focus was on achieving high classification accuracy, we acknowledge the need to test and further enhance the model’s robustness against adversarial inputs. Adversarial training may also be incorporated in future code updates of FungID using techniques like the Fast Gradient Sign Method (FGSM) [46], DeepFool algorithm [47], and Deep Image Flicker (DIF) method in order to evaluate model performance-sensitivity and identify weaknesses [48,49]. By acknowledging and addressing the vulnerability of CNNs to adversarial attacks, we aim to enhance FungID’s robustness and reliability for practical real-world applications, which is of vital importance in fields like healthcare and biotechnology.

Due to the fact that there are no other existing tools up to date specifically designed for fungal identification in silico based on chromogenic profiling, comparative analysis of FungID could only be performed against (1) traditional methods for fungal identification, which rely on examination of morphological features by the human eye, can be time-consuming, and are dependent on expert knowledge [7], or (2) molecular techniques, such as PCR-based methods, which rely on amplifying and analyzing specific DNA sequences to identify fungal species, but require specialized laboratory equipment, skilled personnel, and can be costly or time-consuming [50]. On the contrary, our approach leverages CNNs to automatically analyze and classify fungal species based on spatial patterns in chromogenic profiling, offering a faster and potentially more efficacious alternative. Consequently, while molecular techniques remain the gold standard for fungal identification, also being used here as reference, our pilot work introduces a novel methodology, which combines the efficiency of deep learning with the specificity of chromogenic profiling, offering significant advantages over state-of-the-art methods.

In summary, FungID is very promising in its pilot form by accurately identifying fungal species that affect plants, animals, including humans, or the environment negatively, like *Aspergillus* spp., *Alternaria* spp., or *Fusarium* spp.. The program may identify fungal species, serving as a robust and prompt diagnostic tool with the correct setup. Molecular techniques cannot be replaced as they are the most reliable way to identify fungi; FungID, however, could work as a supplementary tool showcasing high efficiency, cost-effectiveness, and rapid results. Our tool’s ability to provide rapid and reliable identification of these significant fungi can play a crucial role in fungal identification. Despite the promising overall results, several challenges and areas for further investigation remain. The model’s reliance on image quality and preprocessing steps like circle detection underscores the importance of standardizing these processes to ensure consistent performance. Variations in image quality, lighting conditions, and sample presentation can affect the model’s accuracy, necessitating rigorous image preprocessing protocols. Ensuring consistent image quality and robust preprocessing methods is crucial for maintaining the model’s performance across different datasets. The key aspect of the algorithm’s implementation is the use of a GUI, which significantly enhances user accessibility and ease of operation. The GUI allows for real-time monitoring of training progress, parameter adjustments, and direct visualization of results, making the application suitable for everyone in the field. However, it is essential to ensure that the GUI is continually updated to accommodate new features and improvements based on user feedback and advances in machine learning techniques.

## 5. Conclusions

Overall, the proposed algorithm demonstrates significant potential for accurate and efficient fungal species classification. The integration of machine learning techniques with a user-friendly GUI provides a powerful tool for both researchers and practitioners. Future work should focus on addressing identified limitations, increasing the accessibility of FungID by developing its online platform/website, expanding the training dataset, and exploring advanced methodologies to further enhance the model’s performance and reliability. This comprehensive approach will ensure the continued development and refinement of the fungal species classification application, ultimately contributing to improved identification and understanding of fungal species.

## Figures and Tables

**Table 1:** Fungi investigated with chromogenic profiling.

Fungal Species Name	ATHUM ID Number
*Alternaria alternata*	4780
*Aspergillus baeticus*	9539
*Aspergillus caespitosus*	9537
*Aspergillus flavus*	4760
*Aspergillus nidulans*	4381
*Aspergillus ochraceus*	9535
*Aspergillus protuberus*	9532
*Aspergillus sydowii*	9538
*Fusarium sporotrichioides*	4934
*Penicillium chrysogenum*	4813

**Table 2:** Confusion matrix metrics for the examined fungal species in this study. The metrics include Sensitivity, Specificity, Accuracy, Precision/Positive Predictive Value (PPV), Negative Predictive Value (NPV), F1 score, and Matthews’s correlation coefficient (MCC). The average metric results for all species are also provided in the bottom line of this table.

Species	Sensitivity	Specificity	Accuracy	Precision/ Positive Predictive Value (PPV)	Negative Predictive Value (NPV)	F1 Score	Matthews’s Correlation Coefficient (MCC)
*Alternaria alternata*	80.00%	99.51%	99.04%	80.00%	99.51%	0.80	0.80
*Aspergillus baeticus*	20.00%	96.52%	83.61%	53.85%	85.60%	0.29	0.26
*Aspergillus caespitosus*	12.86%	95.94%	81.93%	39.13%	84.44%	0.19	0.14
*Aspergillus flavus*	60.00%	99.75%	98.80%	85.71%	99.02%	0.71	0.71
*Aspergillus nidulans*	100.00%	89.38%	89.64%	18.87%	100.00%	0.32	0.41
*Aspergillus ochraceus*	12.94%	98.18%	80.72%	64.71%	81.41%	0.22	0.23
*Aspergillus protuberus*	97.14%	75.36%	79.04%	44.44%	99.24%	0.61	0.56
*Aspergillus sydowii*	80.00%	86.96%	85.78%	55.45%	95.54%	0.65	0.58
*Fusarium sporotrichioides*	90.00%	99.01%	98.80%	69.23%	99.75%	0.78	0.78
*Penicillium chrysogenum*	100.00%	99.51%	99.52%	83.33%	100.00%	0.91	0.91
**Average results**	**65.29%**	**94.01%**	**89.69%**	**59.47%**	**94.45%**	**0.55**	**0.54**

## Data Availability

The data are included in the article.

## Abbreviations

The following abbreviations are used in this manuscript:

MCC	Matthews Correlation Coefficient
ROC	Receiver Operating Characteristic
PPV	Positive Predictive Value
NPV	Negative Predictive Value
